# Honeycomb-like MnO_2_/Biochar Catalyst Fabricated by High-Energy Electron Beam Irradiation for Degradation of Antibiotics in Swine Urine

**DOI:** 10.3390/biomimetics8010032

**Published:** 2023-01-13

**Authors:** Huan Ma, Zhi Wang, Ling Qian, Gaorui Jin, Pengqi Yang, Dongfang Wang, Shengkai Xu, Dongqing Cai, Zhengyan Wu, Xin Zhang

**Affiliations:** 1 National Engineering Laboratory of Crop Stress Resistance Breeding, School of Life Sciences, Anhui Agricultural University, Hefei 230036, China; 2 Key Laboratory of High Magnetic Field and Ion Beam Physical Biology, Hefei Institutes of Physical Science, Chinese Academy of Sciences, Hefei 230031, China; 3 Key Laboratory of Environmental Toxicology and Pollution Control Technology of Anhui Province, Hefei Institutes of Physical Science, Chinese Academy of Sciences, Hefei 230031, China; 4 College of Environmental Science and Engineering, Donghua University, Shanghai 201620, China

**Keywords:** biochar, microcatalyst, manganese dioxide, HEEB irradiation, degradation, chlortetracycline

## Abstract

The modification of biochar is essential for the development of multifunctional biochar materials with enhanced remediation effects on contaminated water. In this work, a biochar-based microcatalyst with sunlight sensitivity was synthesized by a creative modification method that involved the rapid fabrication of MnO_2_ microspheres by high-energy electron beam (HEEB) irradiation, and loading them into corn straw-derived honeycomb-like KOH-modified biochar (MBC) to obtain a sunlight-sensitive microcatalyst (SSM). The honeycomb-like structure of MBC facilitated the improvement in MnO_2_ dispersion and photocatalytic property through confinement effect. The effects of photocatalyst dosage, initial chlortetracycline (CTC) concentration, solution pH, temperature and coexisting ions on the photocatalytic performance of SSM were systemically investigated. The results indicated that SSM could efficiently degrade CTC in water and swine urine under sunlight, and exhibited high stability against coexistence of urea, Cl^−^ and SO_4_^2−^. Moreover, SSM showed good reusability in regeneration studies. This work provides a novel method for degrading CTC with potential application prospect.

## 1. Introduction

The issue of antibiotics is a key constraint to the sustainable development of ecological and breeding industry. As a typical antibiotic, chlortetracycline (CTC) has been widely used as a growth promoter and antimicrobial agent in pig farms worldwide, due to its broad-spectrum property and low cost [1]. However, excessive intake of CTC in pigs can lead to high levels of CTC and metabolites in pig urine. When released into the environment, pig urine causes soil and water contamination and ecological imbalances, which are then absorbed by crops and livestock, and finally by humans through the food chain [2] and drinking water [3]. Ingestion of CTC may lead to serious human health problems such as arthropathy and antimicrobial drug resistance [4].

In the past decades, although several methods have been developed to remove CTC from water, including adsorption [5,6], coagulation/flocculation [7], and membrane processing [8], secondary contamination and non-degradable antibiotics have limited the application of these methods. Several photocatalysts, such as TiO_2_-based materials [9,10] and SnO_2_-based materials [11], can degrade CTC in water to a certain extent, but the complicated process of material preparation and low sensitivity to visible light also limits their practical applications [12]. Therefore, it is highly desirable to develop a green simple fabrication method to synthesize sunlight-responsive photocatalysts.

As an active photocatalyst, manganese dioxide (MnO_2_) is widely employed to degrade organic pollutants due to its low cost, low toxicity and high sensitivity to sunlight [13,14]. In most reports, MnO_2_ synthesis is used by hydrothermal and co-precipitation methods [15], but these methods require multiple chemicals and complex procedures that are neither economical nor environmentally friendly. On the other hand, the prepared MnO_2_ particles tend to aggregate due to high surface energy and scale effect [16], which greatly affects the photocatalytic performance. Therefore, it is essential to find a suitable carrier to improve the dispersion of MnO_2_ particles. Biochar can be produced by biomass pyrolysis with high porosity and good adsorption properties [17], which may be an attractive candidate for MnO_2_ loading. In addition, compared to the traditional method using reductants, high-energy electron beam (HEEB) irradiation can efficiently and facilely produce highly reactive species in aqueous solution such as reductive solvated electron (e^−^_aq_), hydrogen atom (H·), and oxidized hydroxyl radical (OH·), which reduce MnO_4_^−^ to MnO_2_ nanospheres. Meanwhile, HEEB irradiation is free of secondary pollution and has been widely applied in the field of wastewater treatment. Therefore, this technology can be an attractive and environmental-friendly method to fabricate MnO_2_ because of its high efficiency, low cost and facile property [18]. However, few studies have focused on this point.

In this study, MnO_2_ microspheres were fabricated immediately by HEEB irradiation. Then, MnO_2_/MBC composites were obtained by loading MnO_2_ microspheres into the pores of honeycomb-like KOH-modified biochar (MBC). The degradation performance of MnO_2_/MBC on CTC under different conditions was investigated, the degradation efficiency (DE) of optimal MnO_2_/MBC composites as a sunlight-sensitive microcatalyst (SSM) in swine urine was studied, and the degradation mechanism of CTC by SSM was elucidated. This work provides a facile, green and low-cost method for the preparation of SSM, which is a promising approach to promote the degradation of CTC in swine urine under sunlight.

## 2. Materials and Methods

### 2.1. Materials

Corn straw-based biochar powder (black, bulk density 0.34 g/cm^3^, 100–300 mesh, containing 60% carbon, 35% SiO_2_, and 5% metal (Mg, Ca, and K)) was purchased from Kaidi Electric Power Co., Ltd. (Wuhan, China). The swine urine (2.4% organic matter, including urea, uric acid, and benzoylglycine; about 3–4% inorganic ions, including K^+^, NH_4_^+^, PO_4_^3−^, and Mg^2+^) was provided by Jincheng Pig Farm (Hefei, China). CTC (purity 94%) and other analytical reagents were purchased from Sinopharm (Shanghai, China).

### 2.2. Preparation of MBC, MnO_2_, MnO_2_/BC and MnO_2_/MBC

MBC was obtained by immersing 5 g of raw biochar (BC) powder in 50 mL of aqueous KOH solution (3 mol/L), stirring at 300 rpm for 1 h at 65 °C, followed by four centrifugation-washing cycles and drying at 60 °C for 12 h. The aqueous KMnO_4_ solution (250 mL, 15 g/L) was irradiated with an HEEB accelerator (10 MeV and 10 kW) at a dosage of 30 kGy, followed by four centrifugation-washing cycles and dried at 60 °C for 12 h to obtain MnO_2_ powder after grinding. The given amount of BC or MBC was added to the MnO_2_ solution, stirring for 1 h at room temperature, followed by four centrifugation-washing cycles and drying at 60 °C for 12 h to obtain MnO_2_/BC or MnO_2_/MBC powder (100–200 mesh) after grinding.

### 2.3. Degradation Performance Investigation

A total of 30 mg of MnO_2_/BC (W_MnO2_/W_BC_ = 1:2) or MnO_2_/MBC (W_MnO_2__/W_MBC_ = 1:0, 1:0.5, 1:1, 1:1.5, and 1:2) were added separately into 50 mL of aqueous CTC solution (20 mg/L, pH = 5) at 25 °C. In each case, the suspension was stirred at 350 rpm for 120 min under sunlight, while the control was stirred in the dark with the same conditions. Subsequently, 1.5 mL of the resulting suspension was collected at different intervals and centrifuged at 12,000 rpm for 5 min. The concentration of residual CTC in the suspension was measured by spectrophotometry at a wavelength of 366 nm. Finally, the DE was calculated according to Equation (1):

(1) $$\mathrm{DE}=\frac{\left(C_{t–darkness}-C_{t–sunlight}\right)}{C_{0}}\times 100\%$$

where *C*_0_ was the initial CTC concentration, *C_t__–darkness_* and *C_t__–sunlight_* were the concentrations of residual CTC in the dark and under sunlight, respectively. Based on this, MnO_2_/MBC (at W_MnO2_/W_MBC_ = 1:2) possessed the highest DE value and was thus designated as SSM. All experiments were performed in triplicate. In addition, the influence of pH, the initial concentration of CTC, SSM amount, temperature, and coexisting substances on the aqueous solution DE were also investigated.

### 2.4. Reuse of SSM

SSM (30 mg, W_MnO2_/W_MBC_ = 1:2) was added to 50 mL of aqueous CTC solution (20 mg/L, pH = 5) at 30 °C. After the system was shaken under sunlight for 120 min, the residual concentration of CTC was determined to calculate DE according to Equation (1). Subsequently, SSM was obtained by centrifugation at 12,000 rpm for 5 min, washed 3 times with deionized water and dried in a vacuum oven at 60 °C for 12 h. Finally, the resulting SSM was reused for the degradation of CTC.

### 2.5. Investigation of DE of SygSM on CTC in Swine Urine Aqueous Solution

CTC (1 g) was added to 50 mL of swine urine/water solution (W_swine urine_:W_water_ = 1:40) at pH 5.0 adjusted by 0.1 mol/L of hydrochloric acid. SSM (30 mg) was added to the resulting solution and stirred at 350 rpm for 120 min at room temperature under sunlight or darkness. Subsequently, 1.5 mL of the resulting suspension was removed and centrifuged at 12,000 rpm for 5 min, and the resulting CTC concentration in the supernatant was measured. Finally, the DE of SSM on CTC was calculated according to Equation (1).

### 2.6. Characterization

Scanning electron microscope (SEM) (S-4800, Hitachi Co., Tokyo, Japan) and dispersive X-ray spectrometer (EDX) (Sirion 200, FEI, Hillsboro, OR, USA) were applied to observe the morphology of the samples and measure the distribution of elements in SSM. The crystal and chemical structure of samples were analyzed by X-ray diffractometer (XRD) (TTRIII, Rigaku, Tokyo, Japan) and X-ray photoelectron spectroscope (XPS) (ESCALAB 250, Thermo-VG Scientific, Waltham, MA, USA). The groups of samples were identified using a Fourier transform infrared (FTIR) spectrometer (iS10, Nicolet, Madison, WI, USA). Thermogravimetric analyzer (DSCQ2000, TA, New Castle, DE, USA) was used to perform thermogravimetry-differential thermal analysis (TG-DTA) at temperatures from 25 to 800 °C in an N_2_ atmosphere with a heating rate of 10 °C/min. Brunauer–Emmett–Teller (BET) specific surface areas of materials were measured on an automatic surface area and pore analyzer (Tristar II 3020 M, Micromeritics, Norcross, GA, USA) by physisorption of N_2_ at 77.2 K. CTC concentration was measured using a UV–Vis spectrophotometer (Lambda 365, PerkinElmer, Waltham, MA, USA) at the wavelength of 366 nm. The concentrations of Cl^−^ and NO_3_^−^ were determined by an ion chromatograph (ICS-3000, Dionex, Sunnyvale, CA, USA). The photoelectrochemical properties of the samples were determined using CHI 660E electrochemical workstation (CHI, Shanghai, China). The electron spin resonance (ESR) signal of radical spin-trapped by 5,5-dimethyl-1-pyrroline N-oxide (DMPO) were recorded on a Bruker EPR spectrometer (EMX 10/12, Karlsruhe, Germany). ESR measurement was prepared by mixing the samples in a 40 mM DMPO solution tank (aqueous dispersion for DMPO–·OH and methanol dispersion for DMPO–·O_2_^-^) and irradiated with visible light. CTC samples were analyzed using high performance liquid chromatography (HPLC) instrument (Agilent 1220, Palo Alto, CA, USA) and the degradation products of CTC were identified by a gas chromatography-mass spectrometer (GC–MS, Agilent 7890A-5979C, Palo Alto, CA, USA).

## 3. Results and Discussion

### 3.1. Morphological Observation of BC, MBC, and MnO_2_/MBC

**Figure 1:**
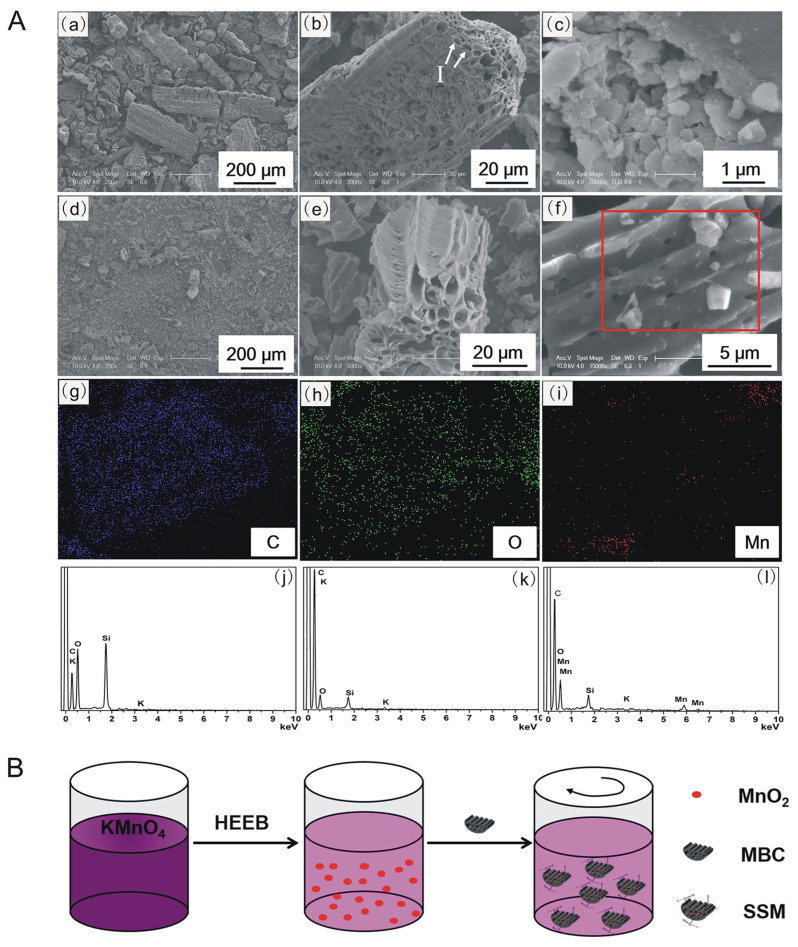
(**A**) SEM images of (**a**,**b**) BC; (**c**) MnO_2_; (**d**,**e**) MBC; (**f**) longitudinal section of MnO_2_/MBC (W_MnO2_/W_MBC_ = 1:2); (**g**–**i**) distribution maps of C, O, and Mn in MnO_2_/MBC (W_MnO2_/W_MBC_ = 1:2); (**j**–**l**) EDX spectra of BC, MBC, and MnO_2_/MBC (W_MnO2_/W_MBC_ = 1:2). (**B**) Schematic illustration of fabrication of MnO_2_/MBC by HEEB irradiation. Note: The area in the red frame of (**A**) (**f**) was used for EDX characterization shown in (**A**) (**g**–**i**).

Figure 1 shows the SEM images and EDX spectra of BC, MBC, MnO_2_ and SSM. Naturally, BC particles with a size of 50–500 μm have a large number of honeycomb-like micropores structures with a diameter of about 1–10 μm (Figure 1A(a)), and some micropores in BC were clogged by fragments (Figure 1A(b) and arrow I). After treating the BC with aqueous KOH solution for 1 h, the prepared MBC particles became smaller (10–200 μm) (Figure 1A(d)), the fragments were removed, and the initially clogged pores were opened (Figure 1A(e)). The modification mechanism might be that KOH reacted with SiO_2_ in BC to form silicate (2KOH + SiO_2_ = K_2_SiO_3_ + H_2_O) [19,20] and tended to be washed away by water. As seen from the EDX spectra, the relative peak intensities of Si and O in MBC were significantly lower than those in BC (Figure 1A(j,k)). According to the EDX analysis (Figure 1A(j,k)), the weight percentages of Si and O in BC decreased from 22.64% and 38.59% to 7.5% and 23.21%, respectively, due to KOH treatment. This removal of SiO_2_ could make BC smaller, unblock the clogged pores, and provide more adsorption sites for CTC adsorption and loading of MnO_2_ in the pores. Figure 1B shows the schematic diagram of MnO_2_/MBC fabrication by HEEB irradiation. HEEB irradiation can induce the generation of a large number of reductive particles such as H and e_aq_^−^ in water [18], thereby reducing KMnO_4_ to MnO_2_. The resulting MnO_2_ particles had a size range from 0.5 to 1 μm (Figure 1A(c)) and tended to aggregate to form large aggregates, which was detrimental to their catalytic performance. Compared to the MBC pores, MnO_2_ particles exhibited smaller sizes and tended to load in the pores; therefore, some MnO_2_ particles could be seen in the longitudinal section of MnO_2_/MBC (Figure 1A(f)), which could also be confirmed by the distribution plots of C, O, and Mn of MnO_2_/MBC (Figure 1A(g–i)). The presence of Mn in MnO_2_/MBC also provided the evidence for successful loading of MnO_2_ in MBC (Figure 1A(l)). Importantly, due to the confinement effect of MBC micropores, the resulting MnO_2_/MBC exhibited higher dispersion than MnO_2_ alone, which potentially promoted the catalytic behavior.

### 3.2. Degradation of CTC by MnO_2_/MBC in Water

**Figure 2:**
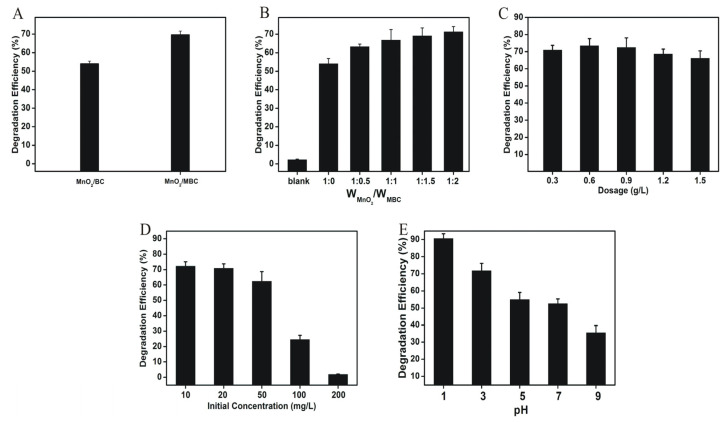
DE of different samples on CTC in water after sunlight irradiation for 120 min at room temperature. (**A**) MnO_2_/BC (W_MnO2_/W_BC_ = 1:2) and MnO_2_/MBC (W_MnO2_/W_MBC_ = 1:2) (dosage of 0.6 g/L, initial CTC concentration of 20 mg/L, pH = 3); (**B**) MnO_2_/MBC with different weight ratios (dosage of 0.6 g/L, initial CTC concentration of 20 mg/L, pH = 3); (**C**) SSM with different dosages (initial CTC concentration of 20 mg/L, pH = 3); (**D**) SSM with different initial CTC concentrations (dosage of 0.6 g/L, pH = 3); (**E**) SSM under different pH (dosage of 0.6 g/L, initial CTC concentration of 20 mg/L).

Figure 2 illustrates the degradation performance of MnO_2_/MBC on CTC I n water under sunlight. Figure 2A shows that the DE of MnO_2_/MBC (W_MnO2_/W_MBC_ = 1:2) is higher than that of MnO_2_/BC (W_MnO2_/W_BC_ = 1:2), which might be due to the smaller size and larger pores of MBC compared to BC, so it was favorable for MnO_2_ Loading. This result also indicated that MnO_2_/MBC had a good response to sunlight.

Figure 2B shows that the DE of MnO_2_/MBC (W_MnO2_/W_MBC_ = 1:0.5, 1:1, 1:1.5, or 1:2) was higher than that of MnO_2_ alone at a given dosage, which proved that MnO_2_ was photocatalytically active to sunlight and MBC as a carrier could effectively enhance the photocatalytic capacity of MnO_2_ to CTC. Moreover, the DE of MnO_2_/MBC increased with the ratio of W_MnO2_/W_MBC_, and the DE of W_MnO2_/W_MBC_ reached a maximum of 1:2 (approximately 73%), which suggested that the combination of MnO_2_ and MBC was beneficial to the degradation of CTC. Therefore, when the ratio of W_MnO2_/W_MBC_ was 1:2, it was the optimal substance for the degradation of CTC and designated as SSM. The influence of SSM dosage on DE was also investigated. As shown in Figure 2C, the DE reached a maximum at SSM dosage of 0.6 g/L and decreased with increasing at SSM dosage >0.6 g/L; therefore, 0.6 g/L was determined as the optimal SSM dosage. Figure 2D shows that the DE decreased when the initial concentration of CTC exceeded 20 mg/L; therefore, 20 mg/L was chosen as the optimal initial concentration of CTC. As shown in Figure 2E, the DE decreases as the pH of the CTC aqueous solution increases from 90.7% (pH = 1) to 33.5% (pH = 9). This might be because acid conditions could promote the production of free radicals, including OH, ·O_2_^−^, and HO_2_ by MnO_2_ under sunlight. These free radicals played key roles in degradation of CTC, which will be discussed and demonstrated in Section 3.3.

**Figure 3:**
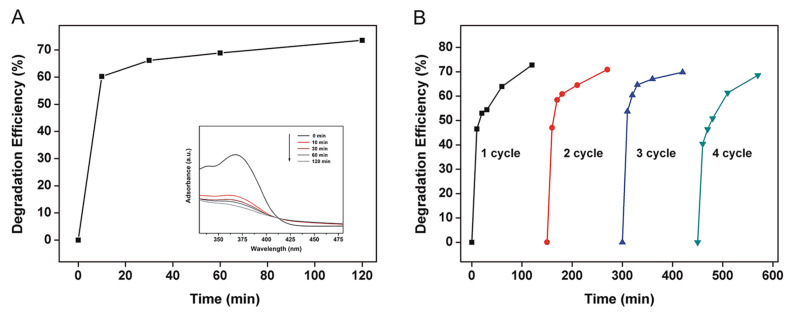
(**A**) DE of SSM on CTC in aqueous solution; (**B**) reusability of SSM for degradation of CTC in aqueous solution. The inserted Figure shows the UV–Vis spectra of CTC aqueous solution treated with SSM.

Figure 3 shows the degradation performance of SSM to CTC and its photocatalytic stability. According to Figure 3A, the DE of SSM increases rapidly from 0 to 60.2% in just 10 min and slowly to 73.5% from 10 to 120 min. The result indicated that SSM had high CTC degradation efficiency and the peak intensity of the UV–Vis absorption spectra of SSM-treated CTC decreased with time (insert of Figure 3A), and the trend was similar to that of DE. In addition, Figure 3B shows that no significant decrease occurred to the DE of SSM after four cycles, suggesting that SSM had good reusability on CTC degradation.

**Figure 4:**
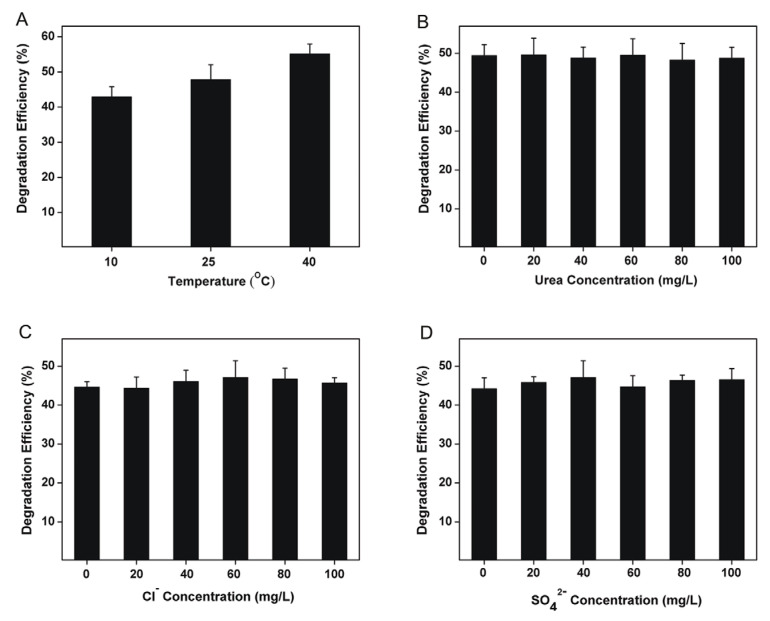
DE of SSM (0.6 g/L) on CTC in aqueous solution (pH = 5, 20 mg/L) under different conditions: (**A**) different temperature; (**B**–**D**) coexistence of urea, Cl^−^, and SO_4_^2−^.

The effects of temperature and coexisting substances (urea, Cl^−^, or SO_4_^2−^) on the degradation of CTC by SSM were also investigated. To simulate the actual CTC-containing water, all experiments were performed at pH of 5. Figure 4A shows that the DE of SSM gradually increased from 10 to 40 °C, which could be explained by the higher migration speed and contact rate between SSM and CTC at higher temperature. After degradation with SSM (0.6 g/L) at 25 °C for 2 h, the concentrations of Cl^−^ and NO_3_^−^ in the resulting solution were determined by ion chromatograph to be 1.318 and 0.37 mg/L, respectively. Based on this, it was calculated that SSM degraded about 47.8% of CTC, which was inconsistent with the result in Figure 4A. In addition, according to Figure 4B–D no significant change was found in the DE of SSM, indicating that the coexistence of urea, Cl^−^, or SO_4_^2−^ had little effect on CTC degradation process using SSM. This result implied that SSM displayed a high stability against urea, Cl^−^, and SO_4_^2−^, and thus had a good application prospect.

In addition, the degradation efficiency of SSM was compared with previously reported TiO_2_-based catalysts and other representative catalysts, including pure TiO_2_ [21], Au-TiO_2_ [21], pristine porous TiO_2_-NS [22], TiO_2_-NS/Pt (TPGA) [22], the commercial CuO nanoparticles [23], and CuO-based photocatalysts [23]. Although the experimental conditions varied slightly, most of these materials showed DE of CTC between 8% and 70% after irradiation for 90–120 min. As discussed above, SSM could reach 60% DE in 10 min and 73.5% in 120 min; thus, SSM was superior or at least comparable to other materials in terms of DE of CTC.

### 3.3. Interaction Analysis

**Figure 5:**
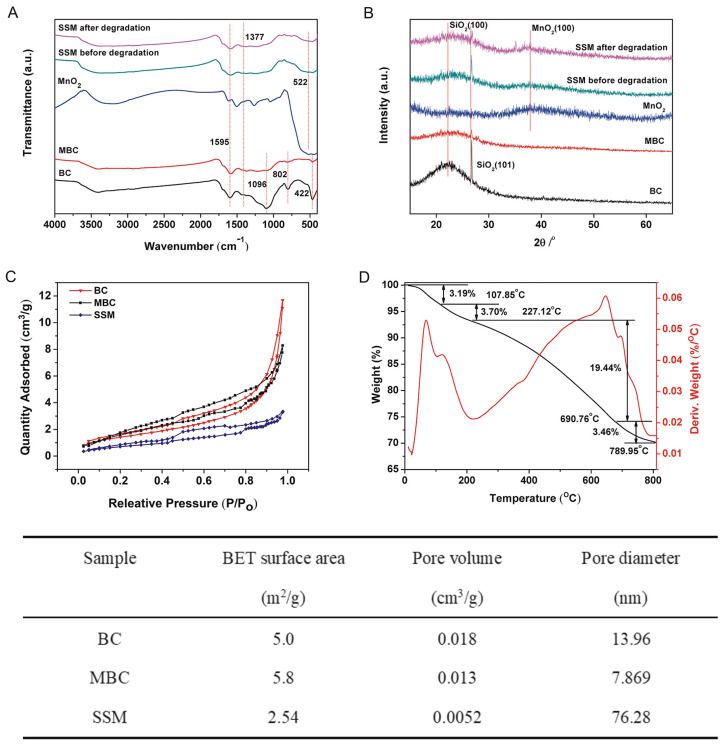
(**A**) FTIR spectra and (**B**) XRD spectra of BC, MBC, MnO_2_, and SSM before and after CTC degradation; (**C**) nitrogen adsorption−desorption isotherms of BC, MBC, and SSM; (**D**) TGA pattern of SSM. The table shows the BET results of BC, MBC, and SSM.

The FTIR spectra of BC, MBC, MnO_2_ and SSM before and after degradation are presented in Figure 5A. The presence of SiO_2_ was evidenced by the characteristic peaks assigned to Si–O–Si flexural vibration, symmetrical stretching and asymmetric vibration at around 422, 802, and 1096 cm^−1^ that could be observed in spectrum of BC [24]. However, for MBC, these corresponding peaks became weaker due to the removal of SiO_2_ by KOH treatment, which was inconsistent with the EDX spectrum (Figure 1A(k)). Additionally, both the characteristic peaks of MBC (C–O stretching peak at 1377 cm^−1^ and aromatic C–C stretching vibration at 1595 cm^−1^) [24,25] and MnO_2_ (Mn–O stretching vibration at 521 cm^−1^) [26] could be found in SSM before and after CTC degradation, indicating that MnO_2_ combined successfully with MBC. Compared to SSM before degradation, no significant structural change was observed in FTIR spectra of SSM after CTC degradation, indicating that SSM had a rather stable structure. This result implied that SSM could act as a stable catalyst with good reusability, which was also demonstrated in Figure 3B. In addition, the broad peak of –OH (~3400 cm^−1^) in the MBC spectrum became weaker after loading MnO_2_ in the SSM spectrum, which might attribute to the formation of hydrogen bonds between MBC (–OH) and MnO_2_, indicating that –OH on the surface of MBC or on the inner surface of micropores contributes to the loading of MnO_2_.

The crystalline structures of BC, MBC, MnO_2_ and SSM before and after degradation were characterized using XRD analysis (Figure 5B). The peaks of SiO_2_ in MBC (22.0° and 26.6°) were less visible, and the half-peak width of the peak at 22.0° expanded from 14.7° (BC) to 24.9° (MBC), which was mainly caused by the partial removal of SiO_2_ by KOH [25] which was in good agreement with the FTIR result (Figure 5A). In addition, both the peaks of SiO_2_ in MBC (22.0° (100) and 26.6° (101)) and MnO_2_ (37.1° (100)) could be found in the spectrum of SSM, which indicated the successful combination of MnO_2_ and MBC. Compared to pre-degradation SSM, the new peaks around 35.06° and 36.76° in post-degradation SSM were mainly attributed to the degradation products of CTC, which were absorbed by SSM surface.

The nitrogen adsorption–desorption isotherms of BC, MBC and SSM were shown in Figure 5C. Based on the N_2_-BET method, the specific surface area, pore volume and average pore diameter of the three materials were illustrated in the insert table. The results showed that all three materials exhibited typical IV isotherm according to the IUPAC classification, which imply the presence of mesopores [25]. Interestingly, the loading of MnO_2_ reduced the isotherm steepness of SSM H4-type hysteresis loop (P/P_0_ > 0.4), revealing the coexistence of SSM mesoporosity and microporosity. Meanwhile, the BET surface area and pore volume of SSM decreased when MnO_2_ was bound to MBC, while its pore size increased significantly from 7.87 nm to 76.28 nm in MBC. This suggested that the larger pore size might be favorable to enhance CTC degradation by providing SSM with more visible light absorption sites as well as more CTC adsorption sites [27,28].

The thermal stability of SSM was evaluated by TG–DTA analysis (Figure 5D). Four stages of weight loss could be seen in the TG–DTA curve: the first two stages of weight loss (3.19% and 3.70%), from 20 °C to 107.85 °C and from 107.85 °C to 227.12 °C, corresponding to the removal of water adsorbed in SSM [27]; the third stage of weight loss (19.44%) from 227.12 °C to 690.76 °C might attributed to water dehydration tightly bonded to the surfaces of MnO_2_ microparticles [12] and the combustion of organic matter remaining in MBC [29]. The final weight loss (3.46%) from 690.76 °C to 789.95 °C was probably assigned to the decomposition of MnO_2_ [30,31] and the secondary product of MBC. This result indicated that SSM had high thermal stability.

**Figure 6:**
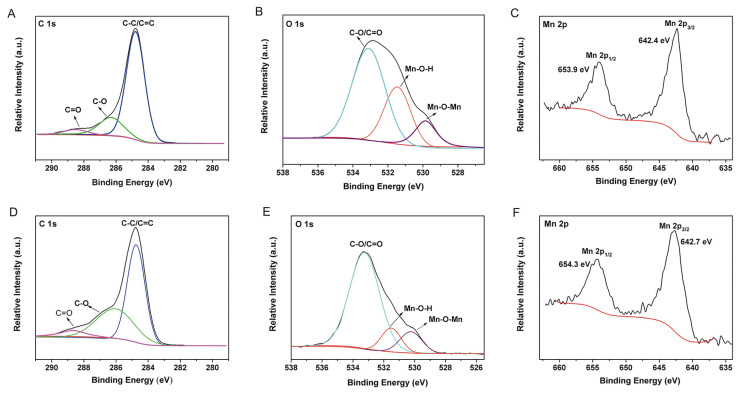
XPS spectra of SSM (**A**–**C**) before and (**D**–**F**) after CTC degradation.

The chemical states of C, O, and Mn in SSM before and after degradation were analyzed using XPS (Figure 6). The results showed that the characteristic peaks of C 1s at 284.6 eV (C–C/C=C), 286.3 eV (C–O), and 288.5 eV (C=O) of SSM did not change significantly after CTC degradation, demonstrating the high stability of these groups (Figure 6A,D) [32]. In addition, as shown in Figure 6B, three O 1s peaks at 529.9, 531.5, and 533.02 eV could be attributed to Mn–O–Mn, Mn–OH, and C–O/C=O bonds, respectively [18]. After the degradation of CTC, the peak at 531.5 eV corresponding to Mn–OH became weak, probably because of the formation of hydrogen bonds between –OH on MnO_2_ and –CONH_2_ on CTC during the degradation process (Figure 6E). The peaks of Mn 2*p*_1/2_ and 2*p*_3/2_ at 653.9 and 642.4 eV assigned to Mn^4+^ [33] did not change significantly after degradation (Figure 6C,F), suggesting that the MnO_2_ in SSM exhibited a good stability during CTC degradation process.

The UV–Vis diffuse reflectance spectra (DRS) of MnO_2_ and SSM were recorded to calculate the bandgap energy of the photocatalyst (Figure S1). The bandgap energy could be estimated by Kubelka–Munk transformation, *ahv* = *A*(*hv* − *Eg*)*^n^*^/2^, where *a*, *h*, *v*, *Eg* represent the absorption coefficient, Plank’s constant, light frequency, and band gap energy, respectively [27]. Based on the above transformation, the *Eg* value of SSM was calculated as 1.75 eV (Figure S1).

### 3.4. Possible Degradation Mechanism Study

The transient photocurrent experiments were conducted on pure MnO_2_ and SSM to study the separation efficiency of photo-generated electron-holes under visible-light. As shown in Figure S2, the photocurrent intensity of SSM was much higher than that of pure MnO_2_, indicating that SSM had higher transfer and separation efficiency of photoinductive carriers, which was mainly due to the confinement effect of MBC that significantly improved the dispersion property of MnO_2_ and facilitated the sunlight adsorption of photocatalysis.

EPR technique coupled with DMPO as a spin-trapping agent was used to detect the reactive oxygen species on CTC in photocatalytic degradation of SSM. As shown in Figure S3, the high-intensity peaks of DMPO–·O_2_^−^ adducts were observed at 10 min of irradiation, while four EPR intensity lines (1:2:2:1) of ·OH radicals could be detected at 20 min. This result demonstrated that the ·OH radicals and O_2_^−^ radicals were the major active species responsible for CTC degradation. When SSM was irradiated by light with energy exceeding the band gap energy, conduction band electrons (e_CB_^−^) and valence band holes (h_VB_^+^) were generated [34,35]. The band gap of MnO_2_ (1.75 eV) was lower than that of TiO_2_ (3.2 eV) [36]. Thus, according to *E* = *hc*/*λ*, sunlight irradiation (wavelength < 1.77 μm) could induce MnO_2_ to produce e_CB_^−^−h_VB_^+^ pairs, and then the generated e_CB_^−^ converted O_2_ adsorbed in MnO_2_ into O_2_^−^, meanwhile, h_VB_^+^ could converted OH^-^ or H_2_O to ·OH by oxidation. When CTC molecules were adsorbed on the surface and in the micropores of MBC, the particles (·OH, O_2_^−^ and e_CB_^−^) of the surface and micropores tended to degrade CTC by redox effect.

**Figure 7:**
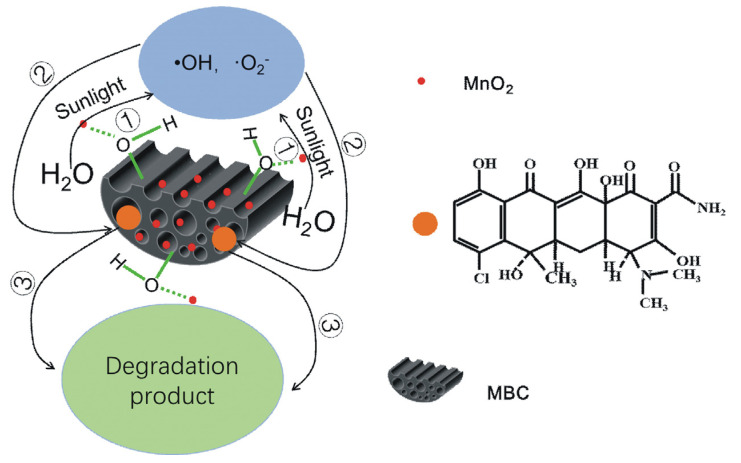
Schematic diagram of CTC degradation process by SSM.

In addition, the CTC samples were analyzed by HPLC (Figure S4) and the degradation products of CTC were identified by GC–MS (Figure S5). As shown in Figure S4A, CTC standard (20 mg/L) were eluted at *t_R_* = 17.40 min with a peak area of about 352.1, while SSM showed no signal UV detection. When the CTC–SSM system was stirred in the dark for 2 h, the peak area of CTC decreased to 268.2, which indicated that 23.8% of CTC was adsorbed on the surface of SSM. After 2 h of sunlight irradiation in the SSM system, no detectable level of CTC could be found, indicating that the residual CTC in this system was completely degraded by photocatalysis, and the final DE of CTC was calculated as 76.2%, which was consistent with the result in Figure 3. The degradation products of SSM on CTC under sunlight were identified by GC–MS, in which mainly consisted of hexadecanoic acid ethyl ester, 9,12-octadecadienoic acid (*Z*,*Z*)- and 13-docosenamide. According to the above results, the possible mechanism of sunlight-sensitive catalytic degradation of SSM on CTC was shown in the schematic diagram in Figure 7.

### 3.5. Degradation Performance of SSM on CTC in Swine Urine

**Figure 8:**
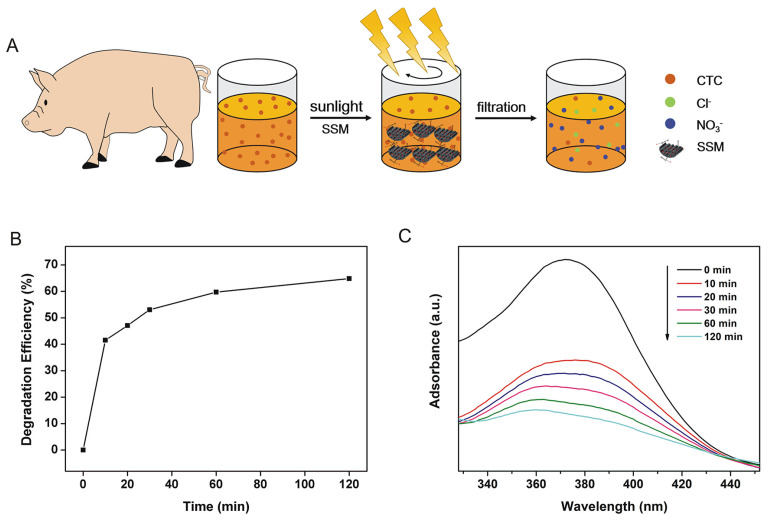
(**A**) Schematic diagram of CTC degradation process in swine urine aqueous solution by SSM; (**B**) DE of SSM (0.6 g/L) on CTC (20 mg/L) in swine urine/water solution with time under sunlight at room temperature; (**C**) UV–Vis spectra of the corresponding SSM-treated CTC swine urine/water solutions.

In practice, CTC-containing swine urine tended to migrate into water to form CTC-containing swine urine aqueous solution. Therefore, it was of importance to evaluate the photocatalytic efficiency of SSM for the degradation of CTC in swine urine. Figure 8A shows the degradation process of CTC in swine urine (W_swine urine_:W_water_ = 1:40) at pH 5.0 under sunlight. Figure 8B shows that DE increased rapidly from 0% to 41.5% in the first 10 min and then increased slowly from 47% to 64.9% (from 20 to 120 min), which was similar to the DE in CTC aqueous solution. The peaks intensity of CTC treated by SSM in UV–Vis absorption spectra also decreased with time, displaying a similar trend to the DE (Figure 8C). This result indicated that SSM could effectively degrade CTC in swine urine aqueous solution under sunlight. Although some previous studies have reported the degradation of CTC by different photocatalysts, few studies have focused on their degradation of CTC in actual wastewater, especially in swine urine. Therefore, this system may have a promising potential to treat CTC in livestock or medical wastewater.

## 4. Conclusions

In this work, MnO_2_ was fabricated immediately using HEEB irradiation and then loaded into the micropores of honeycomb-like MBC to obtain a sunlight-sensitive microcatalyst named SSM. The resulting SSM exhibited higher efficiency on CTC degradation compared to MnO_2_/BC and MnO_2_ due to its honeycomb-like structure, large pore size and the refinement effect resulting from the combination of MnO_2_ and MBC. It exhibited high stability to the coexistence of urea, Cl^−^, and SO_4_^2−^. Moreover, SSM proved to be an efficient microcatalyst with good reusability for CTC degradation in swine urine.

## Data Availability

All data are displayed in the manuscript and Supplementary File.

## Supplementary Materials

The following supporting information can be downloaded at: https://www.mdpi.com/article/10.3390/biomimetics8010032/s1. Figure S1: Plots of (αhν)^n^ versus hν for biochar, MnO_2_ and SSM; Figure S2: Photocurrent response spectra of pure MnO_2_ and SSM; Figure S3: DMPO spin-trapping EPR spectra of SSM; Figure S4: HPLC chromatogram of CTC samples; Figure S5: GC–MS identification of the photocatalytic degradation products of CTC by SSM after sunlight irradiation for 2 h.
