# Supplementary material for: Honeycomb-like MnO2/Biochar Catalyst Fabricated by High-Energy Electron Beam Irradiation for Degradation of Antibiotics in Swine Urine
Source: Biomimetics (Basel). 2023 Jan 13;8(1):32. doi: 10.3390/biomimetics8010032 (PMC9844346; doi:10.3390/biomimetics8010032)
Supplement: Supplementary file 1 [file biomimetics-08-00032-s001.zip › biomimetics-2132711-supplementary.pdf]

Supporting Information

Honeycomb-like MnO_2/biochar catalyst fabricated by high-energy electron beam (HEEB) irradiation for degradation of antibiotics in swine urine

Huan Ma [1], Zhi Wang [1], Ling Qian [1], Gaorui Jin [1], Pengqi Yang [2,4], Dongfang Wang [3], Shengkai Xu [1], Dongqing Cai [3,*], Zhengyan Wu [2,4,*], Xin Zhang [1,*]

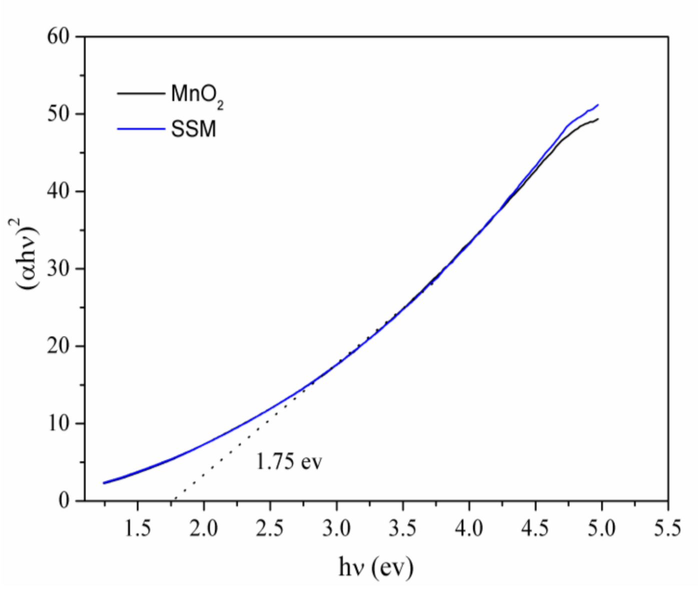

Figure S1. Plots of $(\alpha h\nu)^n$ versus hν for biochar, MnO_2 and SSM

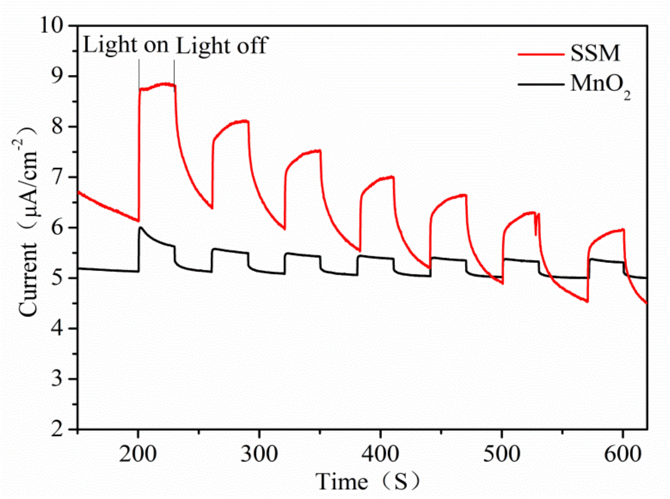

Figure S2. Photocurrent response spectra of pure MnO_2 and SSM

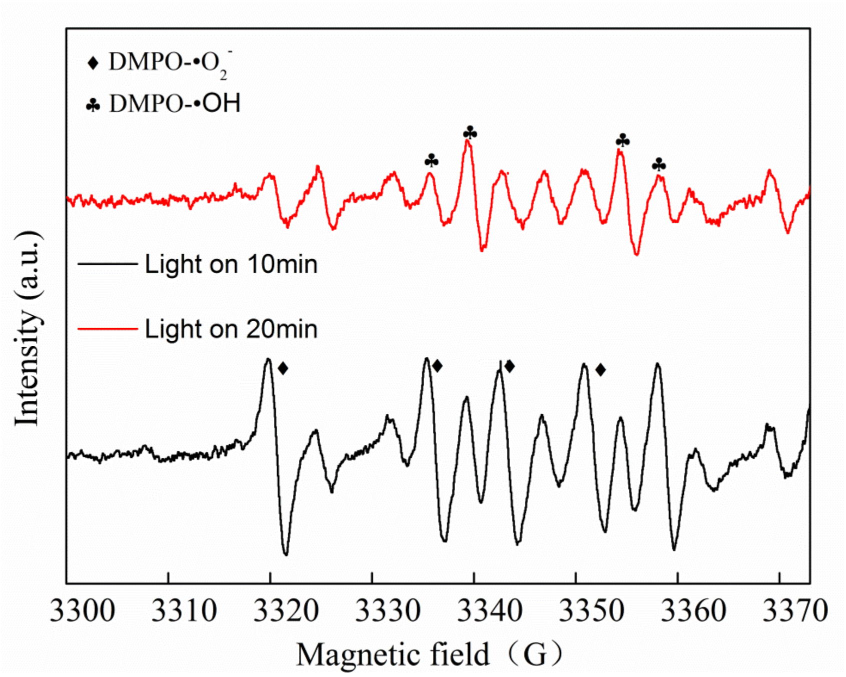

Figure S3. DMPO spin-trapping EPR spectra of SSM

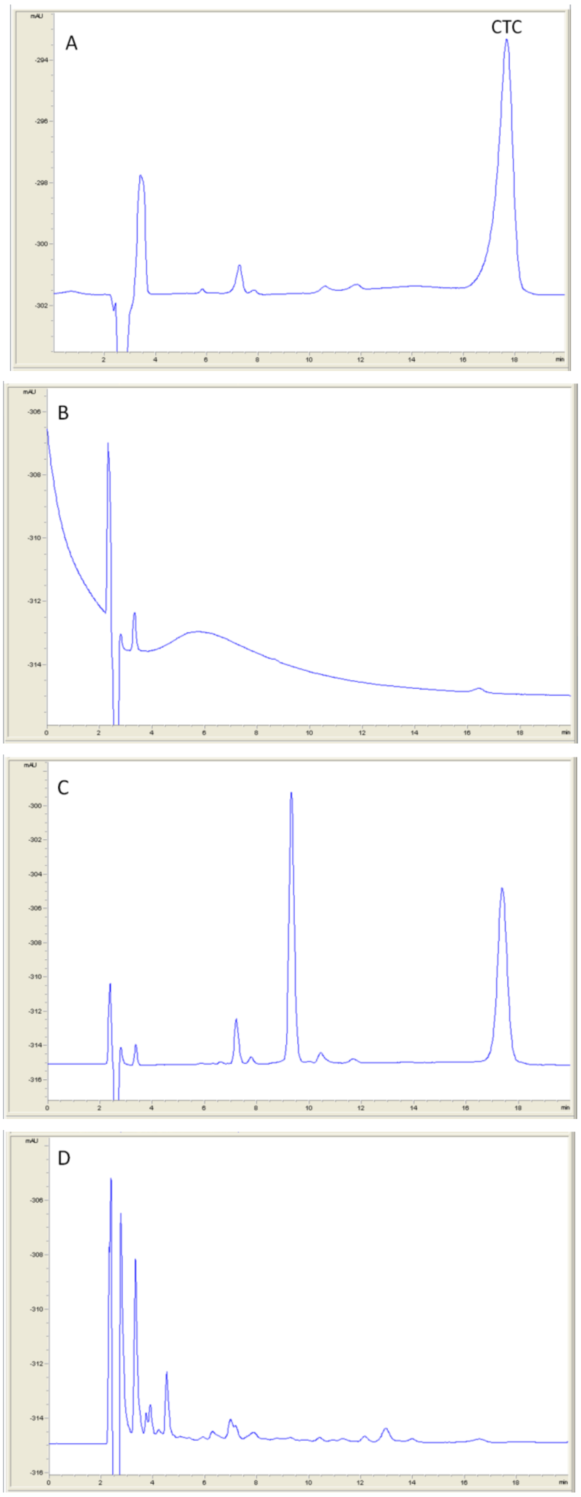

Figure S4. HPLC chromatogram of CTC samples: (A) CTC standard; (B) SSM in aqueous solution ; (C) the sample of CTC-SSM system at 2 h under darkness; (D) the sample of CTC-SSM system at 2 h under sunlight.

HPLC conditions: HPLC instrument (Agilent 1220, USA) equipped with a UV-vis detector. A VF-5ms C18 column (250 × 4.6 mm i.d. stainless steel, 5 μm particles, Agilent Technologies, USA) was used to separate the CTC samples with an isocratic

mobile phase (0.01 M oxalic acid solution:acetonitrile:methanol = 73:17:10, v/v/v) at a flow rate of 0.9 mL/min at 25ºC. The UV wavelength was set at 270 nm, 0.1 AUFS.

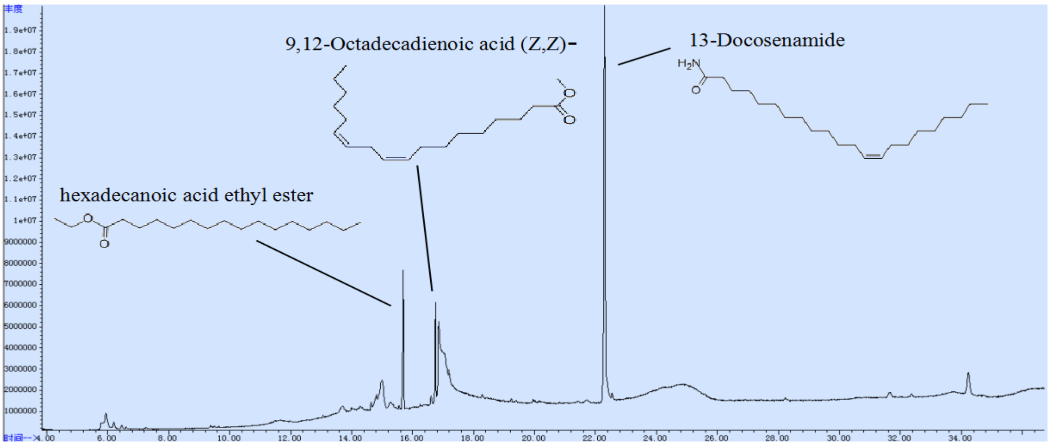

Figure S5. GC-MS identification of the photocatalytic degradation products of CTC by SSM after sunlight irradiation for 2 h.

GC-MS conditions: GC-MS instrument (Agilent 7890A-5979C, USA) with a VF-5ms capillary column (60 m × 0.25 mm, Agilent, USA). The column temperature gradient was programmed from 50 ºC (hold for 2.25 min) to 280 ºC at 20ºC/min and to 290 ºC at 0.5 ºC/min and kept until 35.25 min. The injector volume was 1 μL. Helium was used as a carrier gas with a flow rate of 1 mL/min.
